# Supplementary material for: Does fungal competitive ability explain host specificity or rarity in ectomycorrhizal symbioses?
Source: PLoS One. 2020 Aug 18;15(8):e0234099. doi: 10.1371/journal.pone.0234099 (PMC7433872; doi:10.1371/journal.pone.0234099)

**Fig. S1.** *Pinus strobus* root tips at 20x magnification. (A) Uncolonized. (B) Colonized by *Suillus subaureus*. (C) Colonized by *Suillus americanus*.


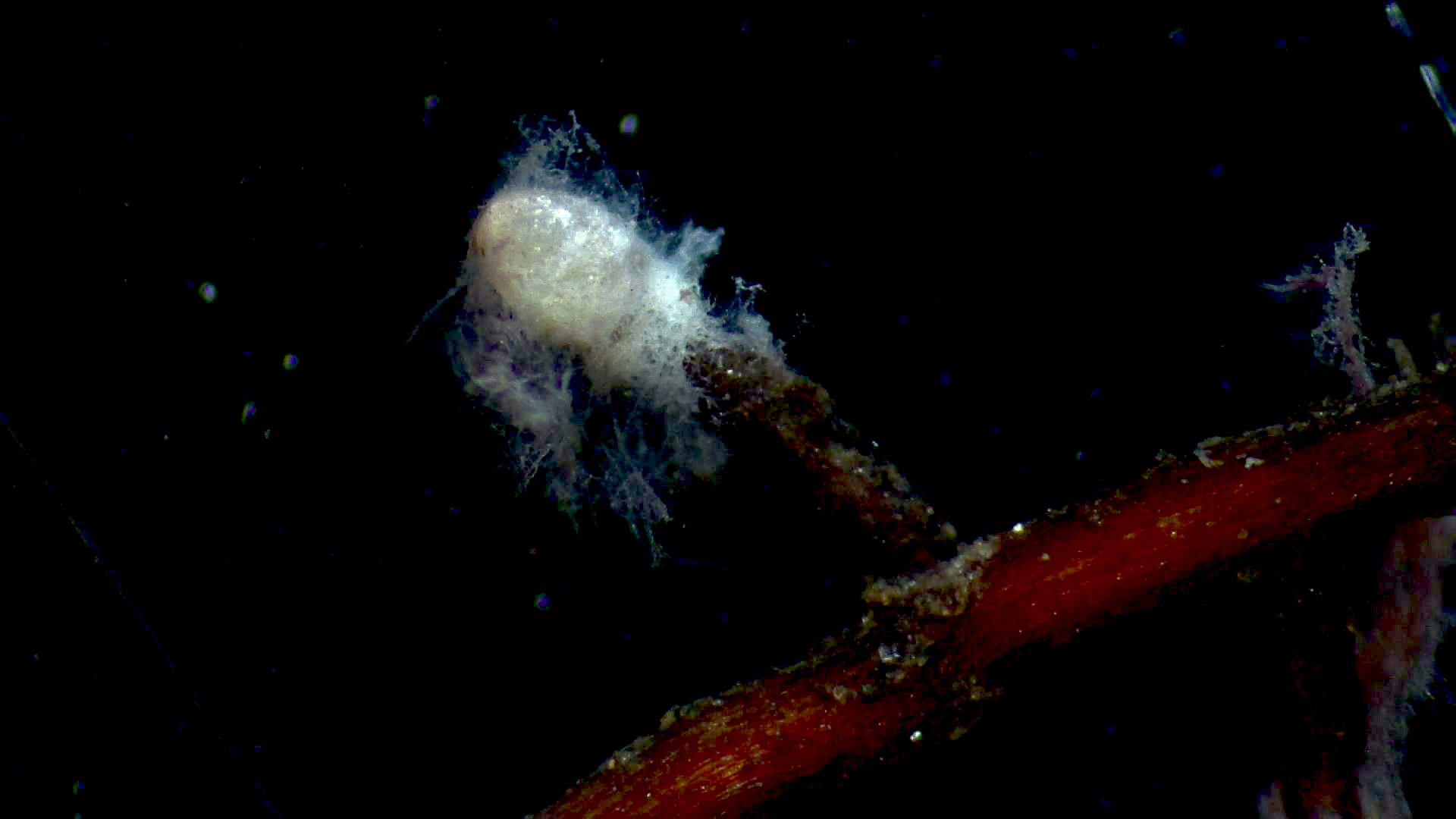

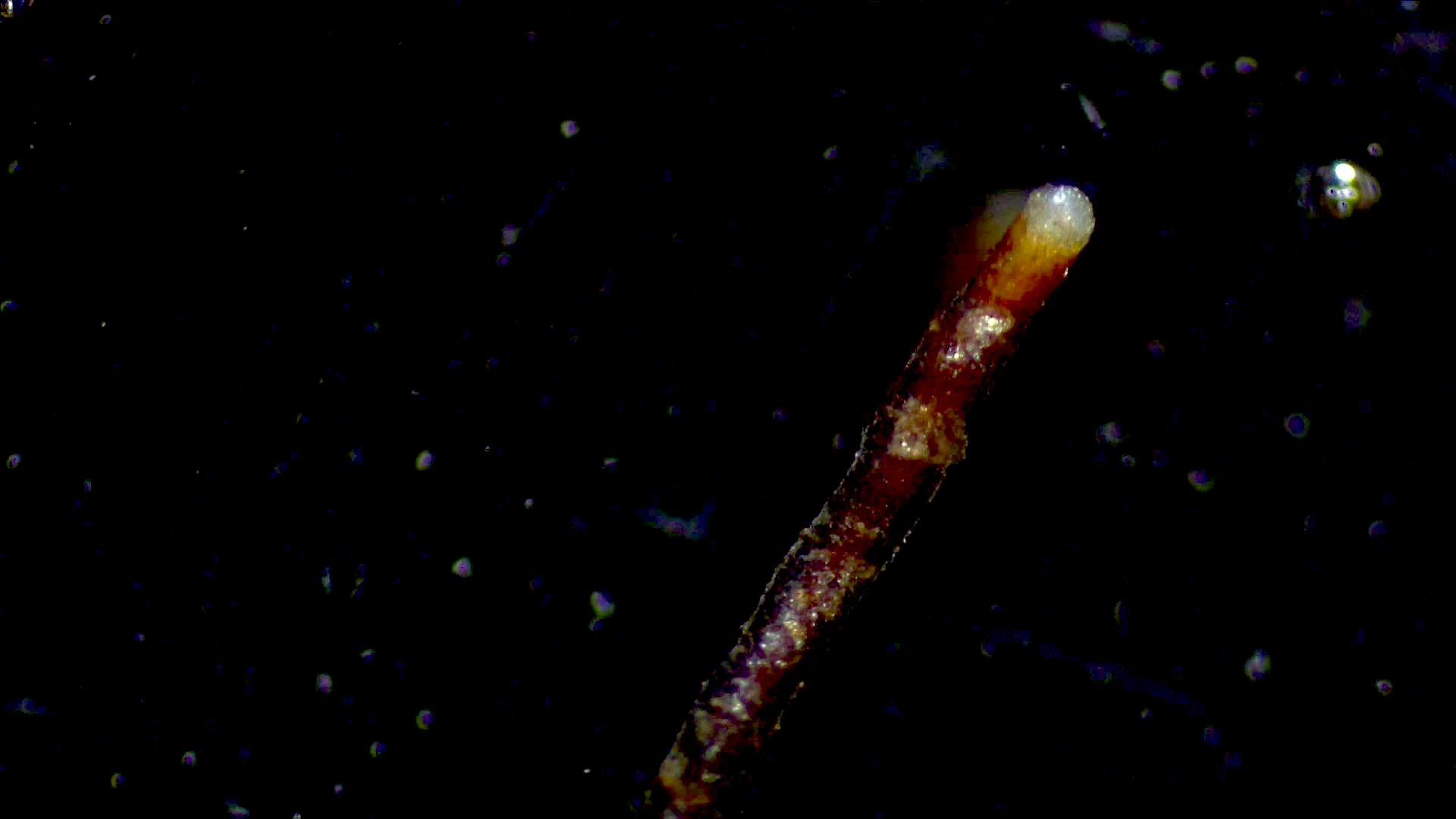


A

B

C


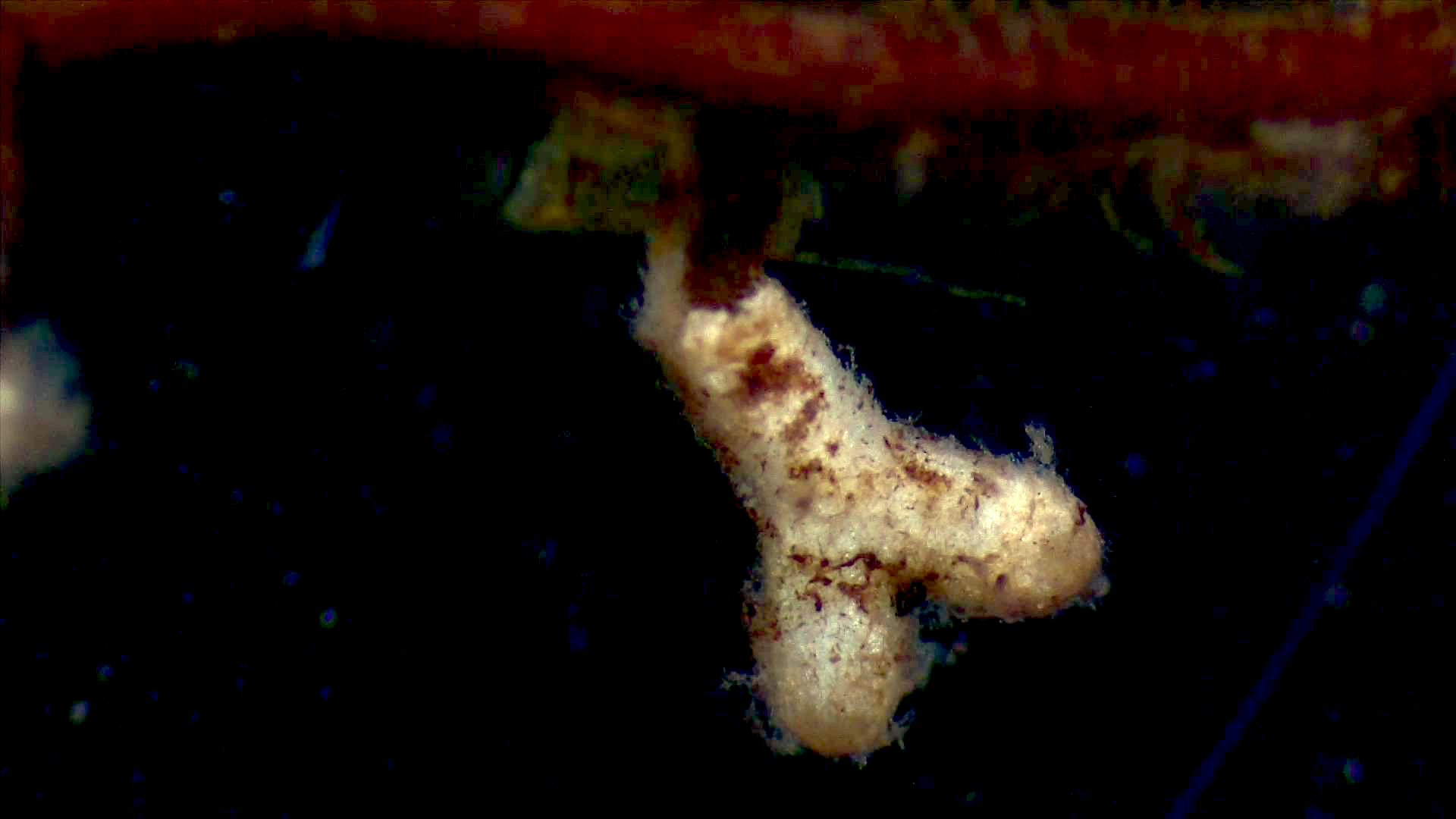

Supplement: S1 Fig — (A) Uncolonized. (B) Colonized by Suillus subaureus. (C) Colonized by Suillus americanus. (DOCX) [file pone.0234099.s001.docx]
