# Supplementary material for: Does fungal competitive ability explain host specificity or rarity in ectomycorrhizal symbioses?
Source: PLoS One. 2020 Aug 18;15(8):e0234099. doi: 10.1371/journal.pone.0234099 (PMC7433872; doi:10.1371/journal.pone.0234099)
Supplement: S2 Fig — (DOCX) [file pone.0234099.s002.docx]

**Fig. S2.** Restriction length fragment polymorphism (RFLP) digest patterns for three *Suillus* species digested using *Alu*1.


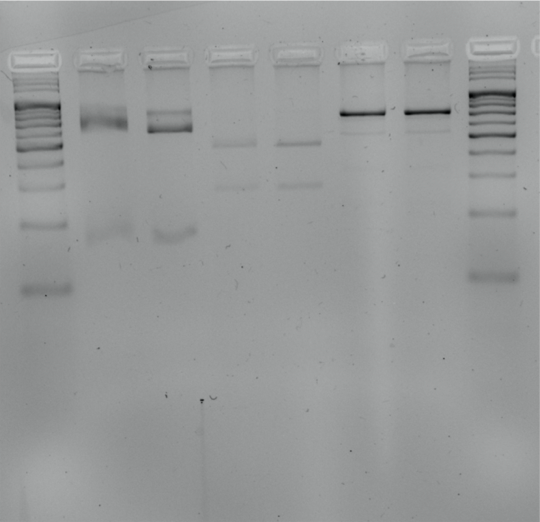


*S. americanus*

*S. subaureus*

*S. spraguei*
