# Supplementary material for: Does fungal competitive ability explain host specificity or rarity in ectomycorrhizal symbioses?
Source: PLoS One. 2020 Aug 18;15(8):e0234099. doi: 10.1371/journal.pone.0234099 (PMC7433872; doi:10.1371/journal.pone.0234099)
Supplement: S3 Fig — (A) Suillus subaureus. (B) Suillus americanus. (C) Suillus spraguei. (DOCX) [file pone.0234099.s003.docx]

**Fig. S3.** Mushrooms of the three *Suillus* species. (A) *Suillus subaureus.* (B) *Suillus americanus*. (C) *Suillus spraguei.*


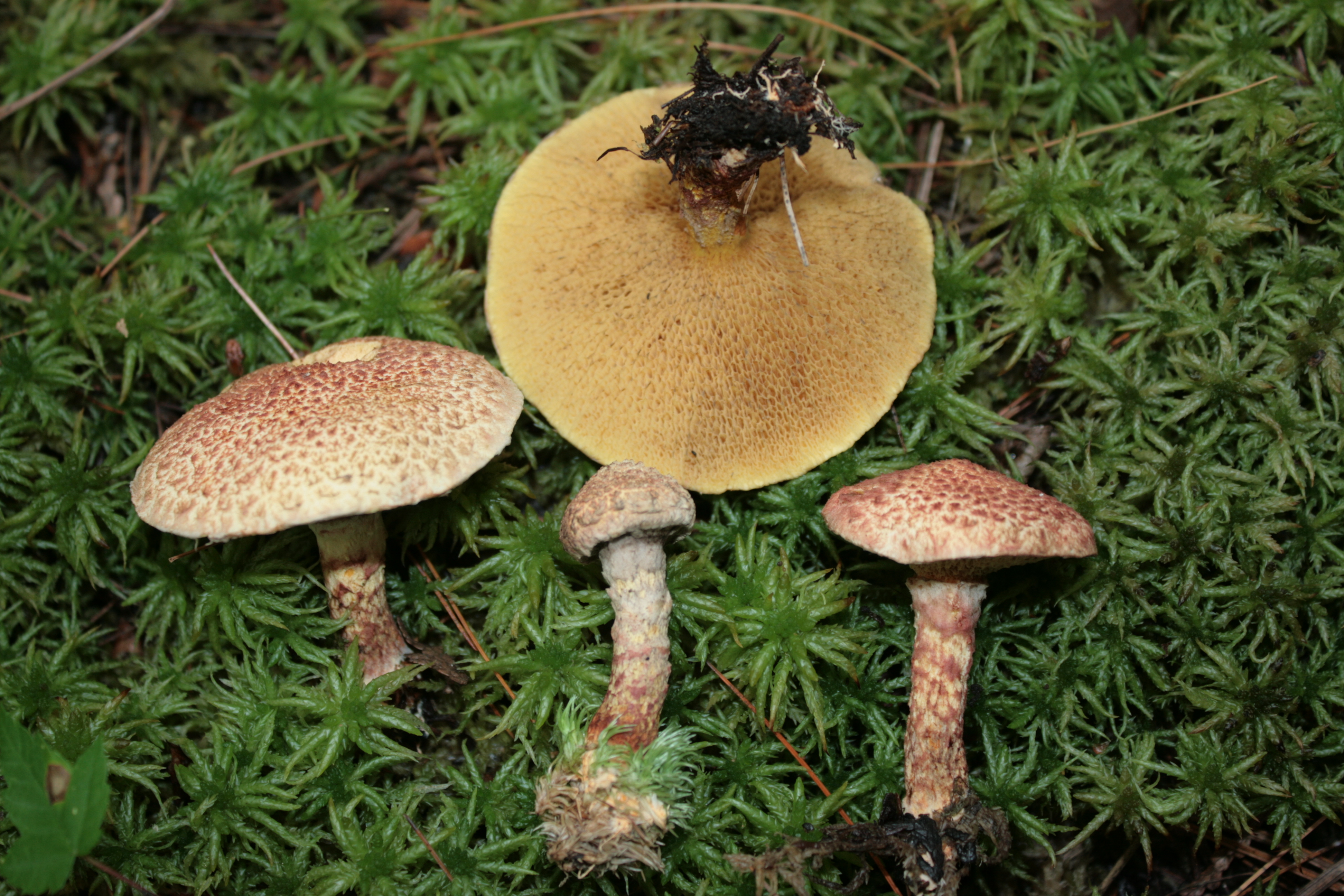

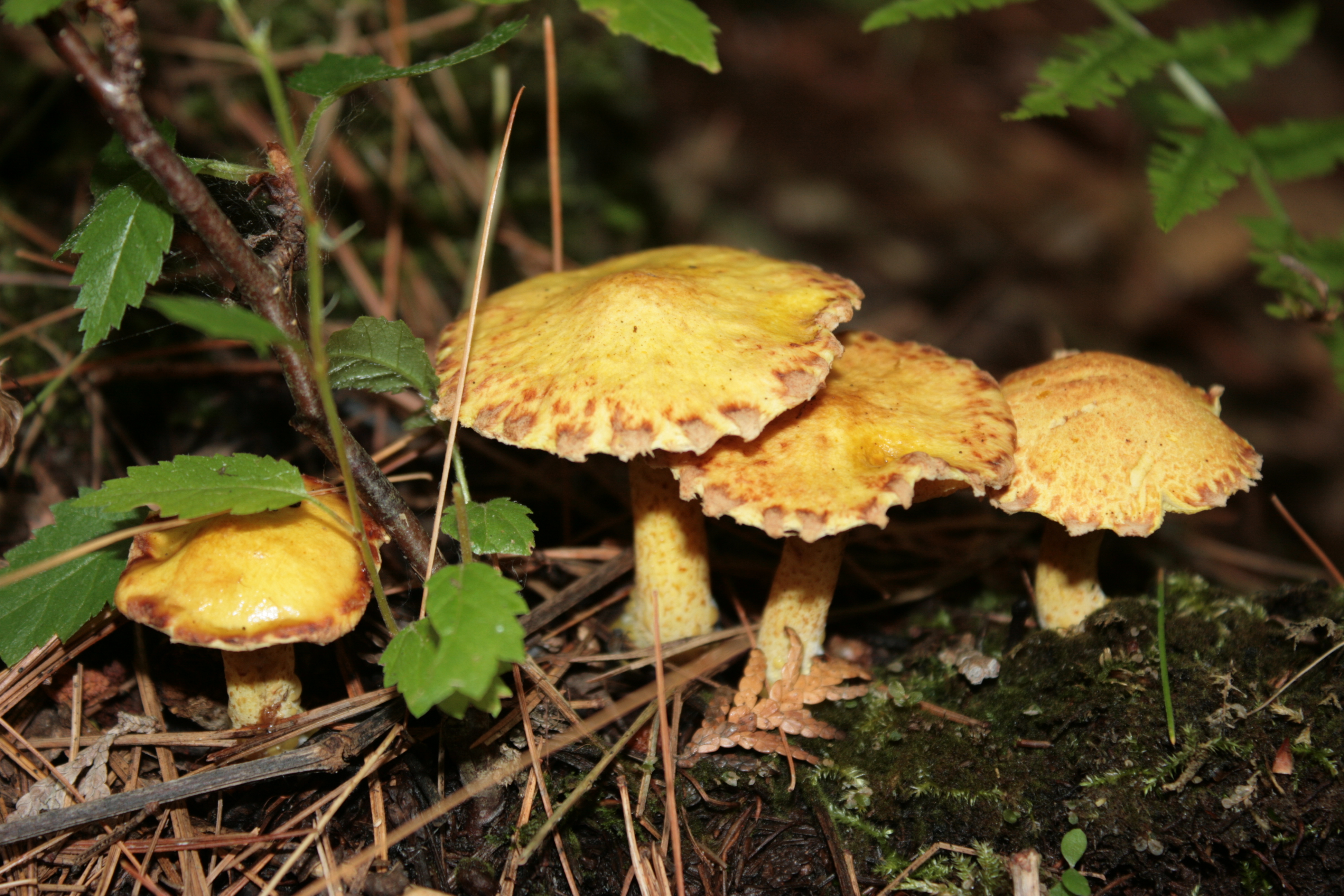

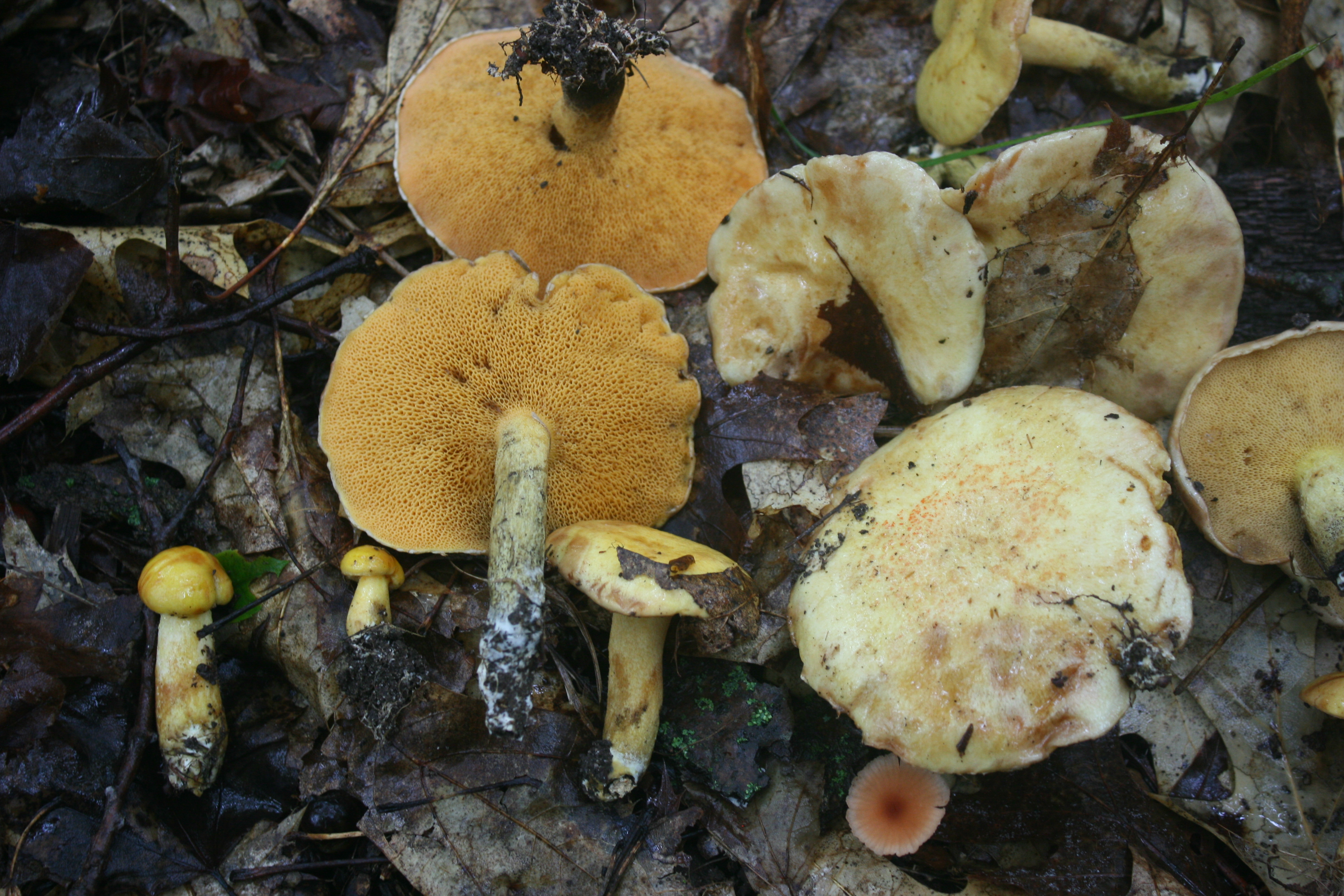


C

B

A
